# Bilateral Femoral Neck Fracture in a Postpartum Woman: Beware of the Risk Factors

**DOI:** 10.1155/2019/4134351

**Published:** 2019-06-24

**Authors:** Ismail Sahan, Julie De Deken, Konstantinos Anagnostakos

**Affiliations:** ^1^Zentrum für Orthopädie und Unfallchirurgie, Städtisches Klinikum Saarbrücken, Saarbrücken, Germany; ^2^Klinik für Allgemein-, Viszeral-, Thorax- und Kinderchirurgie, Städtisches Klinikum Saarbrücken, Saarbrücken, Germany

## Abstract

Bilateral femoral neck fractures pose a rare injury. Literature data describe this entity in association with epileptic seizures, renal osteodystrophy, electric shock, pregnancy-associated transient osteoporosis, and hypocalcemic seizure. In the present work, we report on a rare case of a 28-year-old woman who suffered from such an injury 3 days postpartum. The patient had two predisposing factors (epilepsy history, transient osteoporosis) that were neglected as possible risk factors by the treating physicians. Awareness of the factors might have prevented the emergence of this injury.

## 1. Introduction

Simultaneous bilateral femoral neck fractures are an extremely rare injury. Literature data are scarce about this topic. Single articles have been published, reporting on a possible association with various disorders, such as epileptic seizure [1–3], electric shock injury [4], hypocalcemic seizure [5], and transient osteoporosis during pregnancy [6–8]. Depending on the age of the patient and the type of fracture, either bilateral internal fixation or bilateral total hip arthroplasty have been performed to manage this injury [1–8].

In the present work, we would like to report on an unusual case of a 28-year-old female patient that suffered from bilateral femoral neck fractures three days postpartum.

## 2. Case Report

A 28-year-old woman (1.65 m, 85 kg, BMI 31.2) was referred to our department five days postpartum with a bilateral femoral neck fracture.

During the last two months before the delivery of her first child (begin with the sixth month of gestation), she had complained about increasing weakness and pain in both hips and thighs. In the last two weeks of her pregnancy (begin of the 35^th^ week of gestation), she had to walk on crutches, as she was unable to bear her full weight due to the severe pain in her hips. During this time she took paracetamol daily. Her treating gynaecologist did not initiate any clarification of the symptoms by radiologic imaging methods in this period. Considering her severe pain, it was decided to deliver the child per elective caesarean section (37^th^ +4 week of gestation). Three days postpartum, after being released from the hospital, she had an epileptic seizure and fell. She was admitted to a neurological department of another hospital. During her two-day stay in this department, the woman complained of severe pains in both hips. X-rays and a computer tomography (CT) of the pelvis were performed, revealing bilateral femoral neck fractures. Following this diagnosis, the patient was referred to our department for further treatment.

Regarding her past medical history, she had childhood migraine and epilepsy. She suffered from her first epileptic seizure at the age of 15. She was treated with valproate over 11 years and remained seizure-free during that time. Her treating neurologist stopped the medication two years prior to her pregnancy. The patient did not have any other comorbidities. Particularly, she did not report on any past history of fractures, irregular menstruation, or family history of osteoporosis.

At presentation in our department, the clinical examination showed massively reduced hip movement on both sides because of severe pain. Serum laboratory examination was normal.

**Figure 1:**
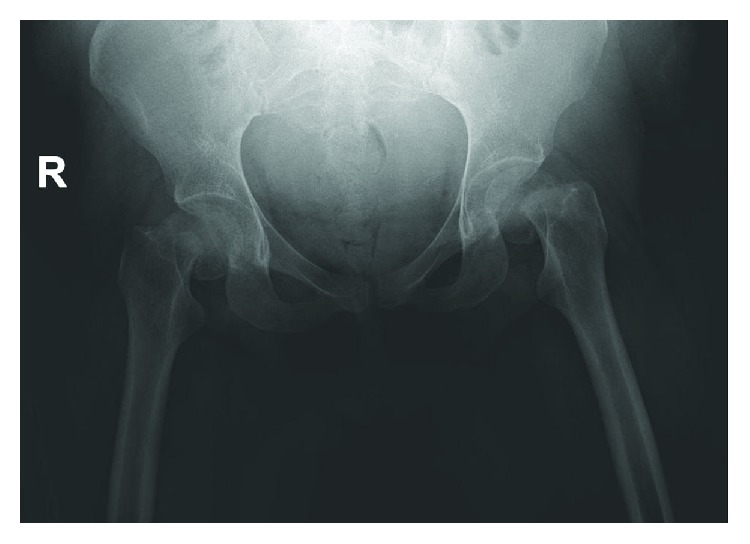
Preoperative anterior-posterior radiographs of the pelvis demonstrating the displaced bilateral femoral neck fractures.

**Figure 2:**
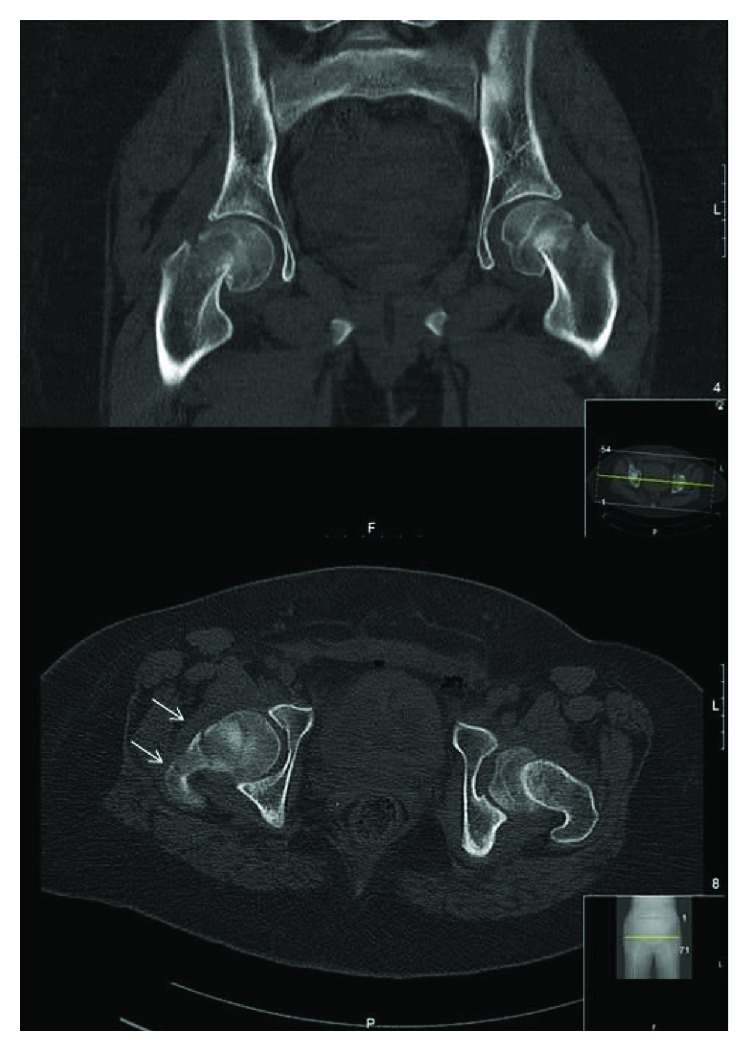
Computed tomography images (coronal and axial plane) confirmed not only the bilateral displacement of the femoral heads but also the presence of a 3-part fracture of the right side (arrows), which helped us to decide for bilateral total hip replacement than internal fixation.

**Figure 3:**
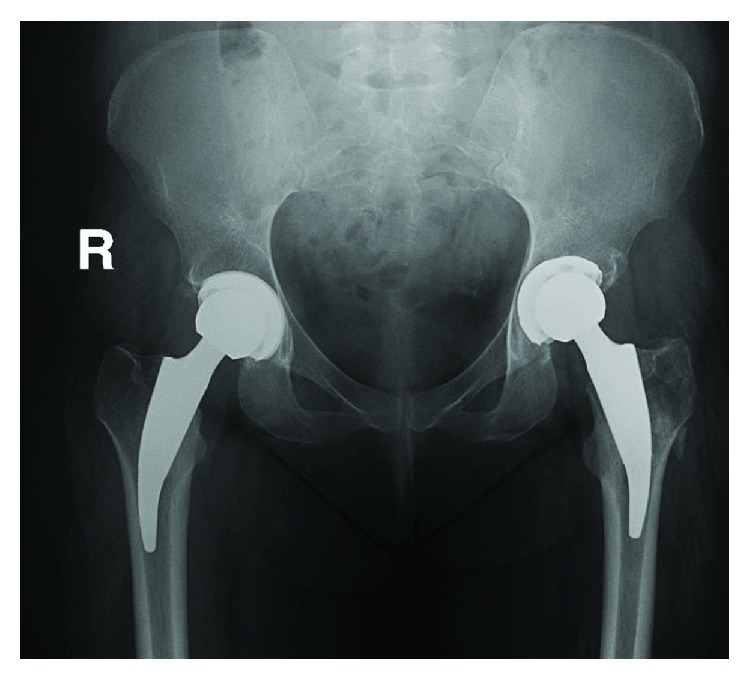
The patient was treated with bilateral implantation of a short-stemmed total hip arthroplasty (Tritanium® cup, Fa. Stryker, Duisburg, Germany; Metha® stem, Fa. Aesculap, Tuttlingen, Germany).

Based on the age of the patient, the limited bone quality, the age of the fractures, and their displacement (Figures 1 and 2), it was decided to perform a bilateral hip joint replacement with the use of short-stemmed prostheses (Figure 3). There were no intra- or postoperative complications. Both femoral heads were sent for further histopathological examination. The results of both femoral heads revealed fracture areas with hemorrhagia, proliferation of fibroblasts in the marrow cavities, and formation of focal woven bone as a sign of the bone remodelling, being indicative for a TOH. Postoperatively, the patient was mobilized on crutches under full weight bearing of the operated extremities. After consultation with our Department of Neurology, the patient restarted her valproate medication. Due to this medication, the patient was advised not to breastfeed her child. Since the TOH is regarded to be a self-limiting disorder, no specific osteoporotic treatment was applied. The further course was uneventful, and the patient was dismissed after two weeks.

At 1-year follow-up, the patient is free of any complaints and has no limitation in the range of motion of both hips (extension/flexion 0°-0°-100°, abduction 50°).

## 3. Discussion

As aforementioned, various predisposing factors have been identified at the site of bilateral femoral neck fractures in young patients [1–9]. Knowledge about these factors and their associated diagnosis and treatment might help preventing the emergence of bilateral femoral neck fractures in some cases.

The interpretation of the history of our patient is more important than the treatment itself in the present work. The patient had complaints over both hips and thighs during the past two months of her pregnancy, a symptom that is very typical for the presence of transient osteoporosis [10, 11]. Transient osteoporosis of the hips (TOH) in pregnancy is an uncommon disease, mostly affecting primipara and women in their third trimester of the pregnancy [12]. Unfortunately, no imaging methods such as magnetic resonance imaging (MRI) were used to clarify these symptoms. The histopathological examination of both femoral heads confirmed this diagnosis. The deposition of thin seams of woven bone in the marrow spaces and the focal mild fibroblast proliferation, as shown in our case, have been also described in other histopathological studies about TOH [13, 14]. From a retrospective point of view, should a MRI have occurred to an earlier time point, the diagnosis of TOH might have been made, and the associated treatment might have been initiated, hence preventing the further weakness of the bone. The second predisposing factor was her positive history regarding epilepsy. Since the birth of child represents additional stress, it might have been advisable to reevaluate her neurological status and restart her antiepileptic medication in order to prevent any seizure before, during, or after the birth of the child.

The presence of two predisposing factors led in our case to the occurrence of the bilateral femoral neck fracture. It could be assumed that the transient osteoporosis enabled the occurrence of this fracture, and the epileptic seizure was the direct cause for that.

Both conservative and operative treatments have been reported in the literature in the management of transient osteoporosis of the hip. Common themes include restriction of weight-bearing activities and analgesia [11]. TOH associated with femoral neck fracture has to be surgically treated. In the absence of avascular necrosis of the femoral head, young patients with a recent fracture can be treated with a closed or open reduction and internal refixation using cannulated screws or a sliding hip screw [11, 15, 16]. Otherwise, hip joint replacement with a total or hemiarthroplasty is necessary [6].

Based on the age of our patient and the type and age of the fracture, we decided to carry out a bilateral total hip arthroplasty. Due to the young age of the patient, short-stemmed prostheses were implanted. This enabled the patient to a quick and pain-free mobilization.

Proximal femur fractures associated with TOH in pregnancy have been reported in several studies [6, 7, 11, 15–19]. However, our literature search showed no reports of femoral neck fracture associated with TOH in patients with epilepsy. To the best of our knowledge, this report represents the first published case where both entities are represented.

A possible differential diagnosis to TOH that has to be born in mind is the pregnancy-associated osteoporosis (PAO). Although the exact etiology is not completely understood, it is assumed that PAO might be the result of a calcium dysregulation during the last trimester based on insufficient maternal intake of calcium and increased resorption due to the rapid mineralization of the fetal skeleton during this time [20]. Typical predisposing factors for PAO involve anorexia nervosa, a long-term low body weight, and a positive family history of osteoporosis [7]. Measurements of the bone mineral density and of the serum vitamin D level are helpful in confirming this diagnosis [7]. The lumbar spine is frequently affected by PAO resulting to vertebral fractures, whereas involvement of the hip joints is regarded to be rare [7, 20].

Although the bone mineral density and of the serum vitamin D level were not determined in our case, we represent the opinion that our patient was rather affected by TOH than PAO. The patient did not have any of the aforementioned predisposing factors, and she did not have any complaints of the lumbar spine but solely of the hip joints. The PAO cannot be surely excluded, but the TOH appears to be the most probable explanation in the present case.

In conclusion, in pregnant women with hip complaints during the last trimester, a MRI should be performed for exclusion of TOH. Should a pregnant have a positive history of epilepsy, the possibly higher risk of a seizure during or after birth should be acknowledged, and consultation with a neurologist is recommended. The presence of both risk factors might be associated with the emergence of bilateral femoral neck fractures, as shown in the present case. Accurate diagnosis and timely initiation of treatment might prevent further complications during or after birth.

## References

[1] Cagırmaz T., Yapici C., Orak M. M., Guler O. (2015). Bilateral femoral neck fractures after an epileptic attack: a case report. *International Journal of Surgery Case Reports*.

[2] Grimaldi M., Vouaillat H., Tonetti J., Merloz P. (2009). Simultaneous bilateral femoral neck fractures secondary to epileptic seizures: treatment by bilateral total hip arthroplasty. *Orthopaedics & Traumatology, Surgery & Research*.

[3] Vanderhooft E., Swiontkowski M. (1994). Bilateral femoral neck fractures following a grand mal seizure. *Annals of Emergency Medicine*.

[4] Shaheen M. A., Sabet N. A. (1984). Bilateral simultaneous fracture of the femoral neck following electrical shock. *Injury*.

[5] Gür S., Yilmaz H., Tüzüner S., Aydin A. T., Süleymanlar G. (1999). Fractures due to hypocalcemic convulsion. *International Orthopaedics*.

[6] Emami M. J., Abdollahpour H. R., Kazemi A. R., Vosoughi A. R. (2012). Bilateral subcapital femoral neck fractures secondary to transient osteoporosis during pregnancy: a case report. *Journal of Orthopaedic Surgery*.

[7] Kasahara K., Kita N., Kawasaki T., Morisaki S., Yomo H., Murakami T. (2017). Bilateral femoral neck fractures resulting from pregnancy-associated osteoporosis showed bone marrow edema on magnetic resonance imaging. *The Journal of Obstetrics and Gynaecology Research*.

[8] Madhok R., Rand J. A. (1993). Ten-year follow-up study of missed, simultaneous, bilateral femoral-neck fractures treated by bipolar arthroplasties in a patient with chronic renal failure. *Clinical Orthopaedics and Related Research*.

[9] Smith M. W., Marcus P. S., Wurtz L. D. (2008). Orthopedic issues in pregnancy. *Obstetrical & Gynecological Survey*.

[10] Hadji P., Boekhoff J., Hahn M., Hellmeyer L., Hars O., Kyvernitakis I. (2017). Pregnancy-associated transient osteoporosis of the hip: results of a case-control study. *Archives of Osteoporosis*.

[11] Lidder S., Lang K., Lee H.-J., Masterson S., Kankate R. (2011). Bilateral hip fractures associated with transient osteoporosis of pregnancy. *Journal of the Royal Army Medical Corps*.

[12] Pallavi P., Padma S., Vanitha Anna Selvi D. (2012). Transient osteoporosis of hip and lumbar spine in pregnancy. *The Journal of Obstetrics and Gynecology of India*.

[13] PLENK H., HOFMANN S., ESCHBERGER J. (1997). Histomorphology and Bone Morphometry of the Bone Marrow Edema Syndrome of the Hip. *Clinical Orthopaedics and Related Research*.

[14] McCarthy E. F. (1998). The pathology of transient regional osteoporosis. *The Iowa Orthopaedic Journal*.

[15] Willis-Owen C. A., Daurka J. S., Chen A., Lewis A. (2008). Bilateral femoral neck fractures due to transient osteoporosis of pregnancy: a case report. *Cases Journal*.

[16] Fokter S. K., Vengust V. (1997). Displaced subcapital fracture of the hip in transient osteoporosis of pregnancy: a case report. *International Orthopaedics*.

[17] Bircher C., Afors K., Bircher M. (2012). Transient osteoporosis of the hip in pregnancy resulting in bilateral fracture of the neck of the femur. *International Journal of Gynecology & Obstetrics*.

[18] Moran M. C. (1995). Iatrogenic Femoral Neck Fracture in Transient Osteoporosis of the Hip. *Clinical Orthopaedics and Related Research*.

[19] Wood M. L., Larson C. M., Dahners L. E. (2003). Late presentation of a displaced subcapital fracture of the hip in transient osteoporosis of pregnancy. *Journal of Orthopaedic Trauma*.

[20] Kovacs C. S., Ralston S. H. (2015). Presentation and management of osteoporosis presenting in association with pregnancy or lactation. *Osteoporosis International*.

